# Colocynth and Colocynthian Reflections

**Published:** 1877-10

**Authors:** James I. Tucker

**Affiliations:** Chicago


### COLOCYNTH AND COLOCYNTHIAN REFLECTIONS.

By Dr. James I. Tucker, Chicago.

It is well known that many of the medicaments in common use at the present day, come down to us as a bequest from antiquity. Among these is one of the *Cucurbitaceæ*, namely, the fruit of the *Citrullus Colocynthis*, which was frequently employed medicinally by the ancient Greeks and the Arabians. It is used at present by the physicians of Great Britain and the United States on account of its drastic-hydragogue properties. It is moreover limited to this use, and these properties are so well understood, that we are obliged to observe the utmost caution in its administration, lest it produce violent griping, nay, more, bloody discharges, inflammation and even death.

Colocynth is administered with us only in the form of pills ; hence, we find as the only officinal preparations in our pharmacopœia, the powder, or solid extract, which forms a constituent of compound pills, or a mass from which pills may be compounded. By a careful observation of the physiological action of colocynth we discover that it has an affinity for the large bowel especially, though it reflexly, or otherwise, increases the general peristaltic movements. The tincture of colocynth is not found among our preparations, but it has for a long time held an honored place in the German pharmacopœia. How it is used by the Germans I am not well informed; but the extreme materialistic views of many of the German physicians, lead me to believe that they would not, as a rule be satisfied unless they should see the feculent discharges, as an evidence that they had accomplished something which would endure the most rankly objective therapeutico-optical scrutiny.

In spite of this, I am going to announce a fact, which I am able to fortify by an array of cases that have come under my personal observation. I state without fear of successful controversion, that *colocynth will allay the pain caused by excessive peristaltic action, better than any drug in use, not excepting opium; providing it be used in the proper dose.* I refer to simple, but nevertheless distressing idiopathic pain, so to speak; pain due to excessive stimulation of the nerves engaged in keeping up the harmonious rhythm of the vermicular movement of the bowels. In such cases I employ not the solid extract, but the *tincture;* and I use the tincture in such small quantities that I expect to meet a large amount of incredulity growing out of *a priori* conclusions. But why, pray, if ipecac in minute doses can allay nausea and vomiting, may not colocynth in small doses allay the very griping which, in large doses, it is capable of producing? I use only just so much of the tincture as to render the excipient—generally water—slightly bitter. In teaspoonful doses, repeated *pro re nata*, I have seen the most speedy relief from very violent griping. Now, since therapeutics is the ultimate aim of classical or humanitarian medicine, I hope that much more attention will be paid hereafter to the hitherto unutilized virtues of drugs which have been supposed to have but a very limited applicability. It will be found that our methods of ascertaining the therapeutical possibilities of drugs are lamentably meagre, and without honest original research we bow too willingly to the shrine of suppositious authority. The truly medicinal properties of many of the drugs in common use lie latent, dormant and neglected, ready at any time to grow and bud and blossom, like the germinal principle, which was at last discovered in the wheat-grains found in the Egyptian catacombs. It is the duty of every practitioner to contribute the results of his experience to the common store of knowledge; not, indeed, to tell us what misery he can occasion by doses of this or that, but how far this or that has contributed, by a careful artistic application to alleviate the sufferings of mankind. The basis of observation has been hitherto very inadequate; but the time is coming, nay, is already here, when the action of drugs may be ascertained with mathematical accuracy. I mean by the neurological method of therapeutics. To this fact, and to the other virtues of the bitter cucumber, which are an illustration of this fact, I now endeavor to call the attention of the medical profession. *Therapeutics, resting on a neurological basis, is to be the therapeutics of the future.*

